# Electro-optic characterization of synthesized infrared-visible light fields

**DOI:** 10.1038/s41467-022-28699-6

**Published:** 2022-03-02

**Authors:** Enrico Ridente, Mikhail Mamaikin, Najd Altwaijry, Dmitry Zimin, Matthias F. Kling, Vladimir Pervak, Matthew Weidman, Ferenc Krausz, Nicholas Karpowicz

**Affiliations:** 1 Max-Planck-Institut für Quantenoptik, Hans-Kopfermann-Strasse 1, 85748 Garching, Germany; 2 Fakultät für Physik, Ludwig-Maximilians-Universität, Am Coulombwall 1, 85748 Garching, Germany; 3 Ultrafast Innovations GmbH, Am Coulombwall 1, 85748 Garching, Germany; 4 CNR NANOTEC Institute of Nanotechnology, via Monteroni, 73100 Lecce, Italy; 5 Present Address: Department of Chemistry, University of California, Berkeley, CA USA; 6 Present Address: SLAC National Accelerator Laboratory, 2575 Sand Hill Rd, Menlo Park, CA 94025 USA

**Keywords:** Terahertz optics, Ultrafast photonics, Optical techniques, High-harmonic generation

## Abstract

The measurement and control of light field oscillations enable the study of ultrafast phenomena on sub-cycle time scales. Electro-optic sampling (EOS) is a powerful field characterization approach, in terms of both sensitivity and dynamic range, but it has not reached beyond infrared frequencies. Here, we show the synthesis of a sub-cycle infrared-visible pulse and subsequent complete electric field characterization using EOS. The sampled bandwidth spans from 700 nm to 2700 nm (428 to 110 THz). Tailored electric-field waveforms are generated with a two-channel field synthesizer in the infrared-visible range, with a full-width at half-maximum duration as short as 3.8 fs at a central wavelength of 1.7 µm (176 THz). EOS detection of the complete bandwidth of these waveforms extends it into the visible spectral range. To demonstrate the power of our approach, we use the sub-cycle transients to inject carriers in a thin quartz sample for nonlinear photoconductive field sampling with sub-femtosecond resolution.

## Abstract (web summary)

A continuum spanning from 300 and 3000 nm is used to synthesize a single-cycle field transient and measure its waveform through electro-optic sampling, speeding up this sensitive technique so that it can access the electric field of visible light.

## Introduction

The observation of ultrafast dynamics requires light fields short enough to both initiate them and clearly probe their evolution. Few-cycle laser pulses in the visible and infrared (IR) spectral range provide ideal conditions for the study of light–matter interaction on the few-femtosecond time scale^1,2^. When focused onto a gas jet, these pulses can produce extreme ultraviolet (UV) bursts through high harmonic generation (HHG)^3,4^. This process results in a spectrum that presents the odd harmonics of the few-cycle pump carrier frequency at lower energies and of a plateau region at higher energies. The last region, when spectrally isolated, can provide attosecond-short pulses whose photon energy and pulse duration depend on the pump’s electric field strength and wavelength. Isolated attosecond pulses have been used to probe atomic-scale electronic motions^4–7^, give new insights into the ultrafast motion of electrons in nanoparticles^8,9^ and complex molecules^10^, revealing new directions for attosecond physics. On the other hand, few-cycle laser pulses continue to prove beneficial in shedding new light on how energy is transferred from photons to charge carriers in solids, on a time scale of just a few hundreds of attoseconds^11,12^. Terahertz spectroscopy has long made use of the electric field waveform for gaining insight into picosecond-to-sub-picosecond carrier dynamics in solids^13–15^. With the advent of optical field metrology, similar investigations of sub-femtosecond dynamics are within reach.

What makes few-cycle pulses so unique, apart from the extreme temporal confinement of energy, is the variety of distinguishable waveforms. The shape of the electric field is strongly influenced by more than just the envelope of the pulse, but also by its relationship with the underlying carrier wave. By changing their relative phase (carrier-envelope phase, CEP—also called global phase in the context of field transients^16^) different pulses with different symmetries can be obtained, as we will illustrate in our system. When the CEP can be actively tuned, the resultant field transients^17–20^ offer an unprecedented degree of control over atomic-scale electronic motions. Fields with different CEPs can induce dramatically different responses of the electronic system of matter, like in the case of molecules where distinct fragmentation pathways can be selected^21^ based on the field symmetry.

The generation of light transients is quite complex, since it requires control over ultrabroad spectra in excess of one octave. Such control poses significant difficulty in the design of optics for dispersion control, such as multilayer optics. Waveform synthesizers mitigate this problem by spectrally splitting the ultrabroadband pulse into different channels^22–26^. This allows the compression of each channel separately and the subsequent recombination into a light transient with sub-cycle time duration. The approach enables the synthesis of electric fields with different shapes by changing the chirp and/or CEP of the pulses transmitted by the individual channels and the relative delay between them with sub-cycle accuracy. The resultant super-octave transients make the carrier-envelope decomposition questionable. Hence, their characterization calls for a direct field detection rather than measurement of the complex envelope, as obtained by pulse measurement techniques such as FROG and SPIDER. Such techniques are able to measure the complex pulse envelope using only the pulse under study, whereas direct field access requires a probing event on the scale of the oscillation period of the electromagnetic wave itself. Attosecond streaking^27^ is a textbook example for probing the electric field of light^28^. It uses the photoemission of an electron by an isolated attosecond pulse as a gate. The necessary generation of attosecond-short probe pulses, however, requires significant pulse energy for the highly nonlinear HHG process. Furthermore, if the synthesized transient itself is used to drive HHG, the parameter space available for pulse synthesis is confined to waveforms simultaneously suitable for the generation of an isolated attosecond pulse in the HHG process^29^.

Electro-optic sampling (EOS) allows the complete characterization of the electric field using a second-order nonlinear process. It has previously been applied to field metrology for frequencies ranging from a few THz^30,31^ through the mid-^32^ and near-IR^33^. The EOS signal is linearly proportional to the field being sampled and the intensity of the sampling pulse. As compared to attosecond streaking, the second-order nonlinearity in EOS, and the use of balanced detection, pave the way towards waveform metrology with the potential to provide an extremely high dynamic range^34,35^. Furthermore, EOS for the detection of waveforms above 100 THz does not suffer from the potential interference of phonon modes in the detection crystal, that often plagues EOS detection at lower frequencies^36^. EOS is easy to implement since it does not require a vacuum environment. As an alternative to EOS, photoconductive antennas^37^ offer a detection sensitivity up to 100 THz^38–40^. Other techniques, often based on photoionization or photoemission to temporally confine the gate, have also shown promise for the detection of fields in the visible range^41–43^. For the detection of weak fields, however, the sensitivity and dynamic range of EOS make it an attractive approach^34,35^, where techniques such as balanced detection can cancel technical noise from the laser. Most importantly for field synthesis applications, the response function of EOS is independent of the CEP of the sampling pulse—as a result, a field synthesis system where the CEP of the synthesized waveform is linked to that of the sampling pulse can be realized while simultaneously keeping the full parameter space available for modification of the generated transient.

In this letter, we report on the extension of the spectral limits of EOS to the visible spectral region—up to 428 THz, from the previous limit of 240 THz^33^. We generate and characterize sub-cycle light transients from a three-channel synthesizer, where one channel, in the UV spectral range, provides the sampling pulse for EOS. We show how the light transients can temporally confine the injection of carriers in quartz, permitting the reconstruction of the waveform of the sampling pulse via nonlinear photoconductive sampling (NPS)^44^. This confirms the versatility of the synthesized fields and the validity of the EOS measurements.

## Results

### Waveform synthesis

**Fig. 1:**
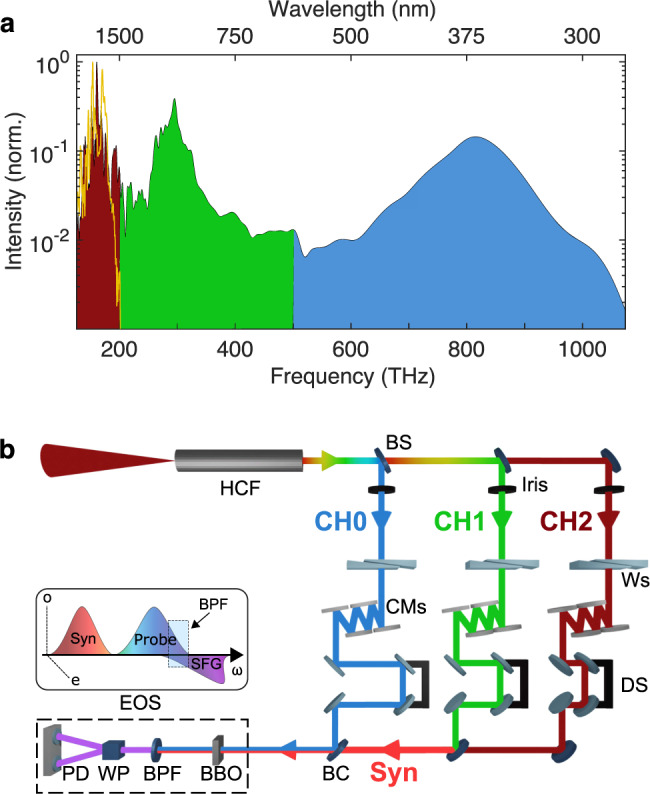
Waveform synthesizer. 15 fs input pulses centered at 1.8 μm, with spectrum is shown in **a** (orange line), are broadened at ambient pressure in an air-filled HCF. The output spectrum spans three octaves (**a**, color-filled spectrum), from the infrared to the ultraviolet. Beam splitters (BS) separate each octave in three different channels (CH0, CH1, CH2), as shown in (**b**). Wedge pairs (Ws) allow the control of the dispersion of each channel separately. Custom-made chirp mirrors (CMs)^55^ are used to compensate for the dispersion in air and through the BS. Temporal and spatial overlap is achieved using delay stages (DS) and beam combiners (BC), respectively. The electric field of the synthesized pulse (Syn) is retrieved via EOS. CH0 and CH1 + CH2 are focused on the EOS crystal (BBO), where sum frequency (SFG) is generated between Syn and CH0 (see inset). The overlapping spectral region between the SFG and CH0 is then isolated using a band-pass filter (BPF, 320 ± 30 nm) additionally improving the signal-to-noise ratio (SNR) of the detection. Finally, the Wollaston prism (WP) splits the band-pass-filtered light into two orthogonally polarized beams which are sent to a pair of balanced photodiodes (PD) for detection.

The generation of ultra-broadband pulses for our synthesizer is achieved by broadening a 15 fs, 0.7 mJ pulse centered at 1.8 μm from an optical-parametric-chirped-pulse amplifier (OPCPA)^45^ (orange line in Fig. 1a) in an ambient-air-filled hollow core fiber (HCF)^46^. The HCF has a length of 33 cm and has an inner core diameter of 250 μm. The HCF output spectrum spans over three octaves (color-filled spectrum in Fig. 1a) and is split into three different channels, approximately one octave per channel, using dichroic beam splitters. The optimal compression of each channel is achieved by using a combination of chirped mirrors with tailored dispersion^47^ and wedge pairs (Fig. 1b). The channels are then recombined with dichroic beam combiners. Since the thickness of the dispersive optics varies between 5 and 15 µm, a stress compensating coating is used on the backside to avoid spatial distortions^48^. The intensity of each channel can be controlled independently, while the temporal overlap can be fine-tuned with delay stages. Finally, the electric field of CH1 and CH2 is reconstructed via EOS.

EOS encodes the information regarding the electric field under study into the polarization state of a sampling pulse. One can picture the process taking place in two steps: first, the lower-frequency portion of the sampling pulse mixes nonlinearly with the wave under study, producing a sum-frequency wave with a polarization orthogonal to the sampling pulse. When the polarization state is measured, this nonlinearly generated wave will interfere with any spectral components of the original probe pulse at the same frequency, which work as a local oscillator for heterodyne or homodyne detection. The inset in Fig. 1b shows that the generated SFG along the extraordinary axis (*e*) of the EOS crystal results in the polarization rotation of the spectral components overlapping with the probe, which is instead oriented along the ordinary axis (*o*). The Wollaston prism (WP) is set at 45° such that the same intensity impinges on the two photodiodes (PD) when only the probe is present. Introducing a time delay between the field under study and the sampling pulse subsequently traces out the electric field of the test wave. This scheme allows for the optimization of both the sensitivity^49^ and spectral bandwidth^33^ of the process through appropriate application of bandpass filters to select the region of optimal spectral overlap. The retrieved waveform is related to the electric field through the spectral amplitude and phase response of the detection system, which depends on the sampling pulse and the phase-matching inside of the crystal. For a detailed description of field retrieval (see the Supplemental Information).

### EOS characterization of NIR-visible waveforms

**Fig. 2:**
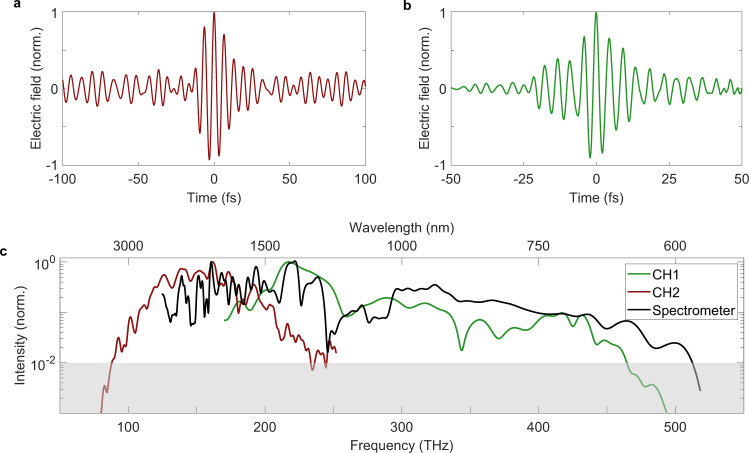
EOS characterization of channels CH1 and CH2. The second (**a**) and first channel (**b**) have been characterized via EOS using CH0 as the sampling pulse. The Fourier transform of the time domain (**c**) shows that frequencies above 400 THz, and therefore in the visible range, can be field resolved via EOS. The black line indicates the spectra of the two channels measured using two spectrometers. The comparison proves that the main spectral features can be reconstructed by the EOS measurements. The gray area (**c**) indicates the region where the SNR is below 1.

In our measurements, the 300–600 nm channel (CH0) is used as a sampling pulse for EOS to retrieve the electric field evolution of the pulses resulting from CH1 and CH2, both separately and combined. The stability, bandwidth, and dynamic range of EOS yield a ratio between the peak intensity and its standard deviation of 12 dB and a dynamic range exceeding 35 dB. Both are primarily determined by the noise characteristics of the laser. A single scan takes around one minute. The time domain traces of CH1 and CH2, within a 100 fs time window, are depicted in Fig. 2a, b, respectively. The full-width-at-half-maximum (FWHM) of the electric field squared is 4.8 fs for CH1 (Fourier limit: 4.2 fs) and 10.8 fs for CH2 (Fourier limit: 7.2 fs). The measured waveforms indicate a nearly optimal compression of the channels, approaching a single cycle. From the measured waveforms of CH1 (Fig. 2b) and CH2 (Fig. 2a), a Fourier transformation was performed to obtain the corresponding spectra (Fig. 2c, red and green), showing that frequencies above 400 THz (750 nm) can be resolved. This demonstrates that the waveform of visible light can be measured with EOS. To verify that our EOS can correctly resolve the spectrum of both channels, two spectrometers have been combined to obtain the spectrum shown in Fig. 2c (solid black line). While the spectrum of CH2 and the red portion of CH1 can be well reproduced, the higher frequencies of CH1 (above 450 THz) differ more from the spectrometer readout. These components are indeed outside of the working range of the CMs, thus, reducing their visibility in the finite time window of the measurement. Another factor that should be considered is that EOS, in the implementation presented here, records the waveform in the center of the focus. The EOS measurement is gated in both space and time, and thus spatiotemporal distortions will result in a difference between the Fourier transform of the measured waveform and the spectrum observed with a grating spectrometer.

### Control of NIR-visible transients

**Fig. 3:**
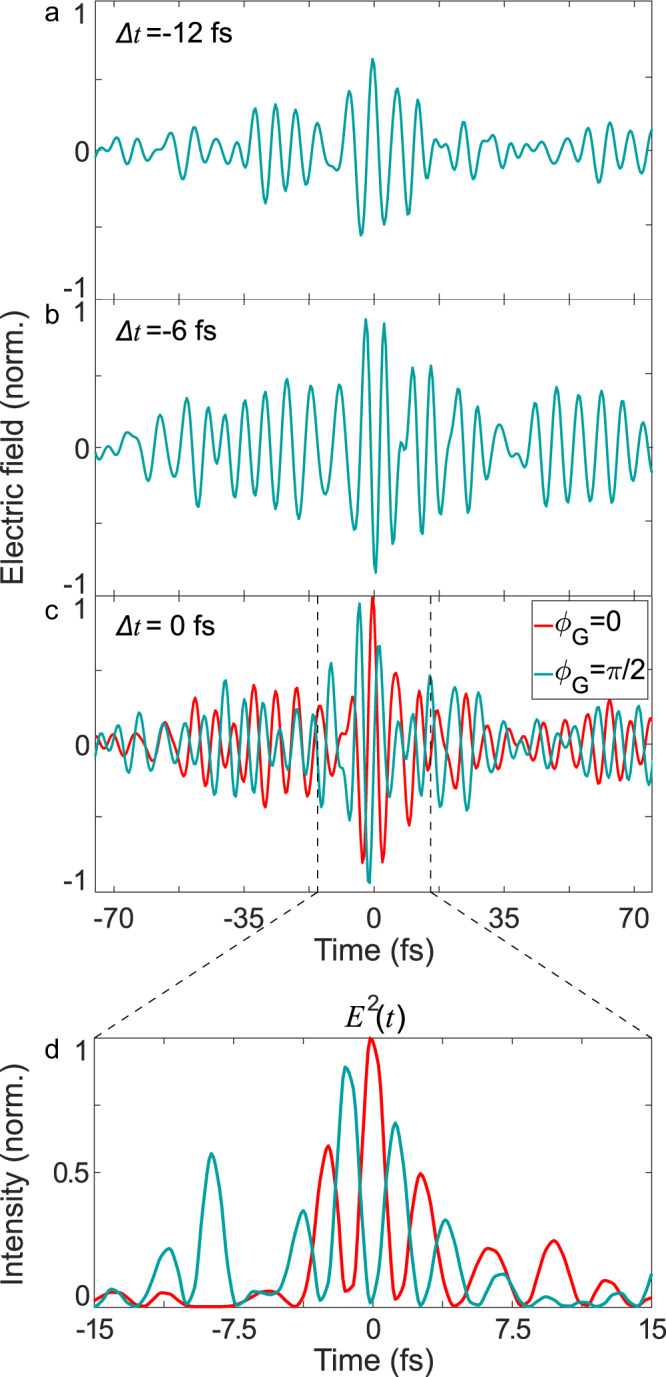
Synthesized waveforms from the combination of CH1 and CH2. From (**a**) to (**c**) synthesized pulses are shown at different relative delays (Δ*t*) between CH1 and CH2. In (**c**) the global phase, *ϕ*_G_, is changed by *π*/2 and the field symmetry is altered accordingly, red and blue trace, respectively. For Δ*t* = 0 light pulses as short as 3.8 fs can be obtained. Panel (**d**) shows the electric field squared of the two traces in (**c**) around the most intense peaks. This illustrates the dramatic impact that changes in the global phase (also known as CEP) can have on the temporal form of the field and intensity.

Channels CH1 and CH2 can be combined into a single pulse to generate super-octave light transients. By controlling the relative arrival time of the pulses in the EOS crystal, different field evolutions can be synthesized (Fig. 3a–c). Pulse durations down to 3.8 fs at 175 THz (1.7 µm) central frequency (wavelength) can be achieved when the arrival time difference (Δ*t*) between the pulses from CH1 and CH2 is set to zero. We note that the electric fields of CH1 and CH2 are robustly synchronized thanks to the negligible timing jitter and drifts that amount to less than 1 fs over 2-h of continuous scanning (see Supplementary Information). Furthermore, an acousto-optic programmable dispersive filter, positioned before the OPCPA, enables the control of the global phase, *ϕ*_G_, of the synthesized pulses (Fig. 3c). This permits the redistribution of the pulse energy as shown in Fig. 3d, where the square of the electric field of the two traces in Fig. 3c is depicted.

Waveform synthesis has already been demonstrated at central wavelengths of 530 nm with four channels^16^ (870, 580, 410, and 300 nm approximate central wavelengths), 780 nm with three channels^23^ (870, 580, and 410 nm), 1.26 μm with two channels^20^ (870 nm and 2.15 μm) and 1.4 μm with two channels^50^ (830 nm and 1.7 μm). Our synthesizer produces pulses at an even longer central wavelength (1.7 μm). This is beneficial for exploiting higher nonlinearities and, in the framework of attosecond metrology, to further push the HHG energy cut-off. Unlike in refs. ^50–53^, the channels are not parametrically amplified. The synthesizer, nevertheless, provides sufficiently energetic and intense pulses (energy ≈ 5 μJ, peak power ≈ 0.5 GW) for activating strong-field phenomena unfolding at field strengths of the order of 1 V/Å in any type of solids, ranging from wide-gap insulators to semiconductors as well as 2D materials.

### Nonlinear photoconductive sampling with synthesized transients

To demonstrate this capability, we employed the sub-cycle transients originating from CH1 and CH2 to temporally confine the injection of carriers in a wide-gap solid, quartz, for non-linear photoconductive sampling (NPS)^44^ of visible–UV fields. NPS utilizes a high intensity injection pulse to launch charge carriers (electrons and holes in the conduction and valence band, respectively) and an orthogonally polarized drive pulse to displace them and thus create a dipole between two electrodes. For a sufficiently sudden carrier injection, the drive electric field can be accessed by measuring the induced-dipole current via an external circuit as a function of the delay between the injection and drive field. To challenge the temporal resolution of the device, we used the output of CH0 as the drive field.

**Fig. 4:**
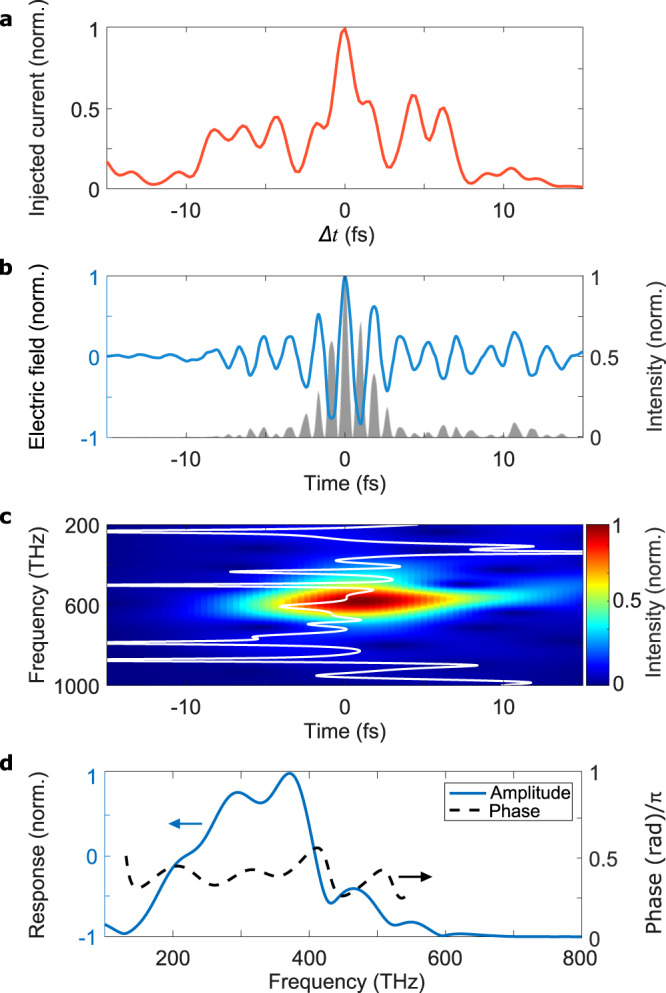
Characterization of CH0 with NPS employing the synthesized transients. To obtain the shortest injection, CH1 and CH2 have to temporally overlap in the NPS sample. **a** The field-induced current is highly sensitive to the relative delay (Δ*t*) between CH1 and CH2. Once the optimum pulse is synthesized (Δ*t* = 0), the reconstructed intensity profile for the pulse in CH0 (**b**) is obtained by injecting carriers with the synthesized pulse and driving them with CH0. The gray area shows the instantaneous intensity of the field, demonstrating that most of the pulse energy is confined within the most intense cycles. The CH0 pulse duration such obtained is 2.8 ± 0.1 fs FWHM, with a Fourier limit of 1.9 fs. **c** shows the spectrogram obtained by Gabor transformation of the trace in (**b**) and the group delay (white line) of the pulse in (**b**). **d** shows the response function of the EOS setup, calculated from the information above (see Supplementary Information for further details).

To optimize the dynamic range and temporal resolution of the NPS device, we measured the number of injected carriers as a function of the relative delay Δ*t* between CH1 and CH2 (Fig. 4a). The maximized carrier injection only tolerates changes of <1 fs in Δ*t* (Fig. 4a), demonstrating the sub-femtosecond control of carrier injection. The most efficient carrier injection is an indication of the best possible temporal confinement (due to the highly nonlinear nature of the generation process). Hence, we utilize the synthesized pulses from CH1 and CH2 that yield maximum carrier injection for sampling the field of the pulses delivered by CH0 (Fig. 4b). The ability to resolve the electric field of UV–visible radiation (CH0), with a period of the shortest frequency of 1 fs, confirms that the injection of carriers is temporally confined to <500.

Beyond this demonstration of temporal confinement, the retrieved electric field of the UV–visible channel (Fig. 4b, blue line) and the corresponding characterization of its spectral phase (Fig. 4c) provide a necessary calibration of CH0 for its application to EOS field measurements of CH1 (Fig. 2b), CH2 (Fig. 2a), and the synthesized transients (Fig. 3). The degree to which CH0 is compressed can be seen by the Gabor transform (Fig. 4c) and the group delay (Fig. 4c, white line); the latter of which indicates the presence of a small amount of residual third-order dispersion. The pulse duration is 2.8 fs, with a Fourier limit of 1.9 fs.

The precise reconstruction of such a broadband sampling pulse is the most challenging and previously unavailable ingredient of optical waveform synthesis. In any waveform measurement technique, the electric field that one wishes to measure is convolved with a detector response function in the process of conversion to the recorded signal. In THz spectroscopy, both signal and reference are convolved with the same function. For evaluating the linear (dielectric) response of the sample, this response function cancels out and is of no concern. By contrast, nonlinear spectroscopy requires knowledge of the true electric field, relying on the detector response function.

Based on a second-order nonlinear effect, EOS is well described by the nonlinear wave equation. When performed in a thoroughly characterized medium such as BBO, the response function may be accurately determined. Unlike attosecond streaking and NPS, the EOS response function is independent of the CEP of the sampling pulse, allowing for the characterization of synthesized fields across all adjustable parameters without affecting the detection. The response function, shown in Fig. 4d, needs to be determined once, and will remain valid for all values of CEP while the synthesized pulse is modified. The CEP-independent response function makes EOS an ideal and easy-to-implement technique for sampling synthesized electric fields.

To make this feasible, the sampling pulse must be precisely known. This, in turn, relies on the ability to synthesize a short enough transient to sample the field with sub-femtosecond time resolution. Thus, the combination of PHz-bandwidth measurement techniques and near-one-femtosecond UV sampling pulses, provides a unique and complete solution to the problem of measuring a synthesized pulse in the IR–visible spectral range.

In conclusion, we have demonstrated that EOS driven by near-single-cycle visible–UV pulses can resolve optical fields up to the visible light spectrum—extending the EOS bandwidth by a factor of two, as compared to the previous record^33^, and demonstrating continuous sensitivity and high fidelity over two octaves. This enabled the controlled synthesis of sub-cycle optical transients covering the spectral range of 700–2700 nm. We have shown that such transients can be used to control and optimize the injection of sub-femtosecond carrier wave-packets in solids, which allow field sampling in the UV range. Multi-octave waveform synthesis combined with ultrabroadband field sampling based on an all-solid-state apparatus provides a powerful new platform for sub-femtosecond control and measurement of nonlinear interactions in solids. Direct field measurements of ultrafast transients will allow for clean and detailed studies of sub-cycle light–matter interaction via attosecond polarization spectroscopy^54^, temporally confining both linear and nonlinear interactions. It also opens new avenues in molecular spectroscopy by the emerging capability to field-resolve not only nuclear^35^ but also electronic wave packet dynamics.

## Supplementary information

Supplementary Information

## Data Availability

All data are available from the corresponding author upon reasonable request.
