# Supplementary material for: Electro-optic characterization of synthesized infrared-visible light fields
Source: Nat Commun. 2022 Mar 2;13:1111. doi: 10.1038/s41467-022-28699-6 (PMC8891359; doi:10.1038/s41467-022-28699-6)
Supplement: Supplementary file 1 — Supplementary Information [file 41467_2022_28699_MOESM1_ESM.pdf]

Supplementary Information for

Electro-optic characterization of synthesized infrared-visible light fields

Enrico Ridente[1,2,5], Mikhail Mamaikin[1,2], Najd Altwaijry[1,2], Dmitry Zimin[1,2], Matthias F. Kling[1,2,6], Vladimir Pervak[1,2,3], Matthew Weidman[1,2], Ferenc Krausz[1,2] and Nicholas Karpowicz[1,4, ✉]

[1] Max-Planck-Institut für Quantenoptik, Hans-Kopfermann-Strasse 1, 85748 Garching, Germany

[2] Fakultät für Physik, Ludwig-Maximilians-Universität, Am Coulombwall 1, 85748 Garching, Germany

[3] Ultrafast Innovations GmbH, Am Coulombwall 1, 85748 Garching, Germany

[4] CNR NANOTEC Institute of Nanotechnology, via Monteroni, 73100 Lecce, Italy

[5] Present address: Department of Chemistry, University of California, Berkeley, CA, USA

[6] Present address: SLAC National Accelerator Laboratory, 2575 Sand Hill Rd, Menlo Park, CA 94025, USA

✉email: nicholas.karpowicz@mpq.mpg.de

Main parameters of the three-channel synthesizer:

CH0 is used as an EOS sampling pulse to characterize the combination of CH1 and CH2. The pulse durations (FWHM of the electric field squared) have been calculated using the traces recorded with NPS for CH0 and EOS for CH1 and CH2. The bandwidth, the pulse durations and their Fourier limit are reported in Supplementary Table 1 below. The measured peak power and pulse energy are 0.5 GW and 5 μJ, respectively. To understand why the conversion efficiency of the synthesizer is low it is necessary to consider that the power of CH1 and CH2 needs to be the same to have the shortest possible pulses. Despite having an efficiency of 60% out of the HCF most of the power goes into CH2, limiting the achievable power of the light transients. Furthermore, the power of the measurable pulses is limited by the damage threshold of the EOS crystal. In order to maintain the ability to measure the true output waveform of the synthesizer in the current configuration, the final pulse energy of the synthesizer is limited to 5 μJ, although significantly more is available as reserve and can be applied by opening the irises in the synthesizer arms (in excess of 50 μJ). Since subtle nonlinear propagation effects can significantly alter the temporal form of a single-cycle transient, any extrapolation beyond what is actually measured can lead to significant errors, and thus the energy stated reflects only the energy of the pulse whose field has been recorded.

Supplementary Table 1.

	**Channel 0 (CH0)**	**Channel 1 (CH1)**	**Channel 2 (CH2)**	**Synthesized (CH1+CH2)**
**Bandwidth**	300 - 600 nm	600 - 1500 nm	1500 - 3000 nm	600 - 3000 nm
**Pulse duration**	2.8 ± 0.1 fs	4.8 ± 0.3 fs	10.8 ± 0.6 fs	3.8 ± 0.2 fs
**Fourier limit**	1.9 fs	4.2 fs	7.2 fs	3.3 fs

Spectrum out of the hollow core fiber:

Waveform synthesizers work by separating and recombining spectral components of an ultrabroad spectrum into tailored fields. A careful characterization of the spectrum is therefore necessary to properly design a synthesizer.

Our system is fed with OPCPA laser pulses (15 fs at 1.8 µm) spectrally broadened in an ambient-air-filled hollow core fiber (HCF, 250 µm inner core diameter), providing an output spectrum spanning over three octaves. The use of ambient air at normal pressure for spectral broadening allows us to avoid the application of additional transmissive optics along the beam path of CH0, which would produce positive group-delay dispersion and thus requiring an additional set of chirped mirrors.

The spectrum, after passing through the HCF, is measured using the setup shown in Fig. 1Sa. The power and the spectrum of each narrowband portion of the output light are obtained by spectrally dispersing the beam with a prism and spectral filtering with a slit. This allows for a measurement of the power-weighted spectrum (Fig. 1b in the main text). The setup also enables switching between two different spectrometers (from 250 to 1000 nm: MayaPro2000, Ocean Optics and from 1000 to 2700 nm: NIRQuest-512, Ocean Optics) for the complete coverage of the three-octave broadband spectrum. The spectral brightness is depicted in Fig. 1Sb showing the normalized power for each wavelength component of the output spectrum. The lower resolution at longer wavelengths is due to the relatively low dispersion of the prism.

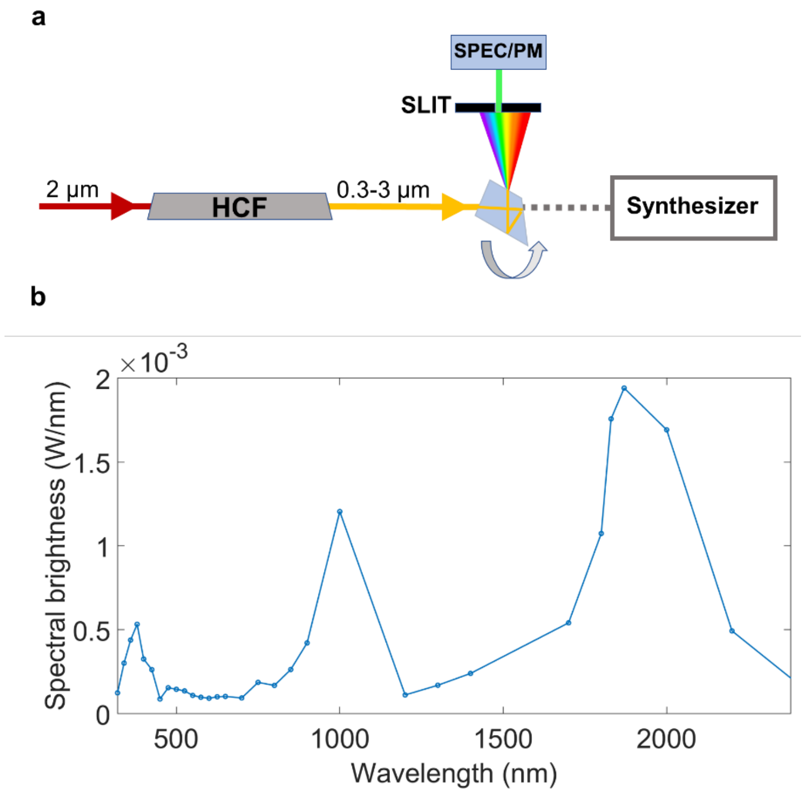

Figure 1S. a, The setup utilized to characterize the three-octave spectrum out of the HCF. The p-polarized beam after the HCF is spatially dispersed by a prism and sent to either a spectrometer or a power-meter after passing through a slit. **b**, Corresponding normalized power measured for different wavelengths of the output spectrum.

Synthesizer stability:

The nonlinear processes before the pulse synthesis couple intensity fluctuations into spectral fluctuations, while mechanical instabilities couple to phase fluctuations. These instabilities can result in the lack of reproducibility of the synthesized waveforms, making the system particularly difficult to apply for highly field sensitive measurements — interferometric stability is required. Being fed by the OPCPA (see main text), any drift of the amplified seed will directly affect the spectral broadening, and eventually, the synthesized fields. To avoid pointing instabilities coming from the OPCPA, a beam stabilization system (4D-Aligna, TEM Messtechnik) is implemented before the HCF to mitigate transverse beam fluctuations. Two motorized mirrors equipped with piezo actuators compensate both slow and fast fluctuations. Before the entrance of HCF, a thin Pellicle window sends 8% of the OPCPA power to two InGaAs detectors. In addition, active temporal synchronization is implemented for the pump and seed of the OPCPA leading to the nearly constant spectrum and the CEP of the OPCPA output (see [28] for further details). The synthesizer stability was measured by continuously recording EOS traces over a time window of two hours (Fig. 2S).

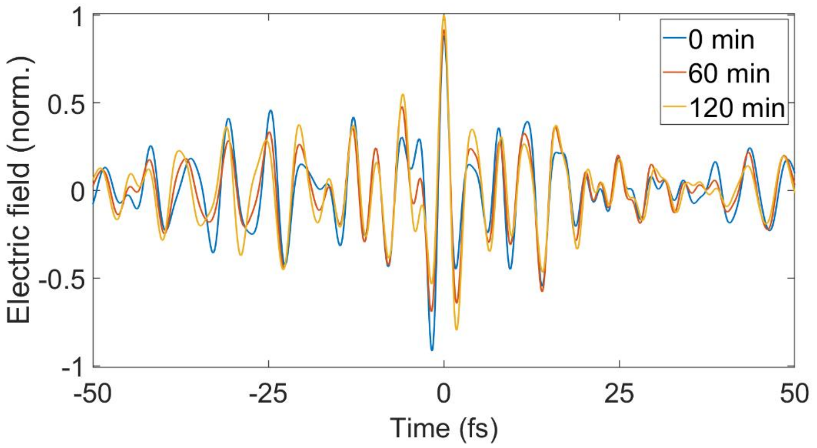

Figure 2S: Long-term stability of the synthesized light transient. The measurements reveal how the waveform preserves its asymmetric profile for more than two hours. The traces shown have been recorded with an interval of 60 minutes.

NPS measurements:

NPS requires a different optimization procedure compared to EOS. In our case, the combination of CH1 and CH2 is used as the injecting pulse. The best compression of these two channels can be achieved by optimizing the injected current in the NPS sample as a function of the pulse compression (Fig. 3S). This can be easily changed by translating one wedge in a pair of glass wedges (Fig. 1c). The readout also provides access to the CEP of the pulses. This implies that both optimal compression and CEP can be controlled by varying the amount of material. CH1 and CH2 need both to be compressed and have the same CEP on the surface of the sample to be able to resolve ultraviolet-visible frequencies in a single carrier-injection event. Once the correct wedge position is found, the highest value of the injected current can be achieved by changing the temporal overlap between the two channels (Fig. 4a).

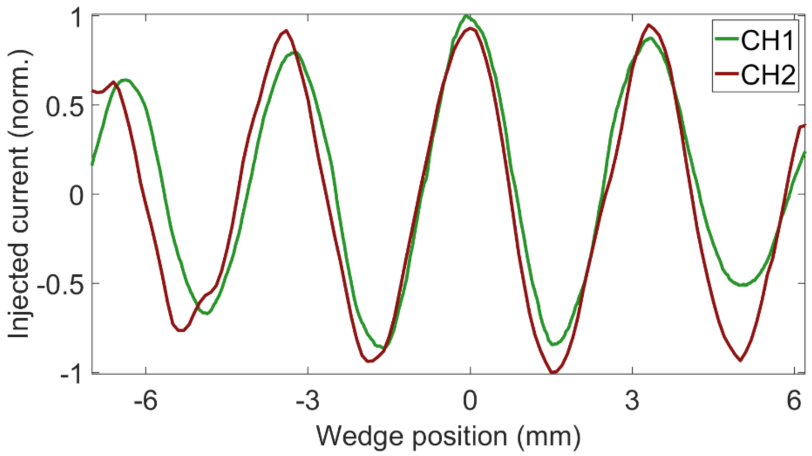

Figure 3S: Dispersion scan of CH1 and CH2. The injected current is optimised by changing the amount of glass along each channel. BaF_2 wedges are used to compress CH1 and SiO_2 wedges for CH2.

Starting from the shortest light transient shown in Fig. 3c (red line), it is possible to calculate the same quantity measured in Fig.4a. For the thin quartz sample used in the experiment, the calculated carrier injection rate (orange line) and the integrated injection (blue line) are shown below in Figure 4S. The trends show how the injection of carriers is strongly confined to the region around the maximum of the synthesized field, providing a reliable readout of the CH0 pulse. The time axis is the same one of Fig. 3c, that is the delay between the synthesized pulse and CH0.

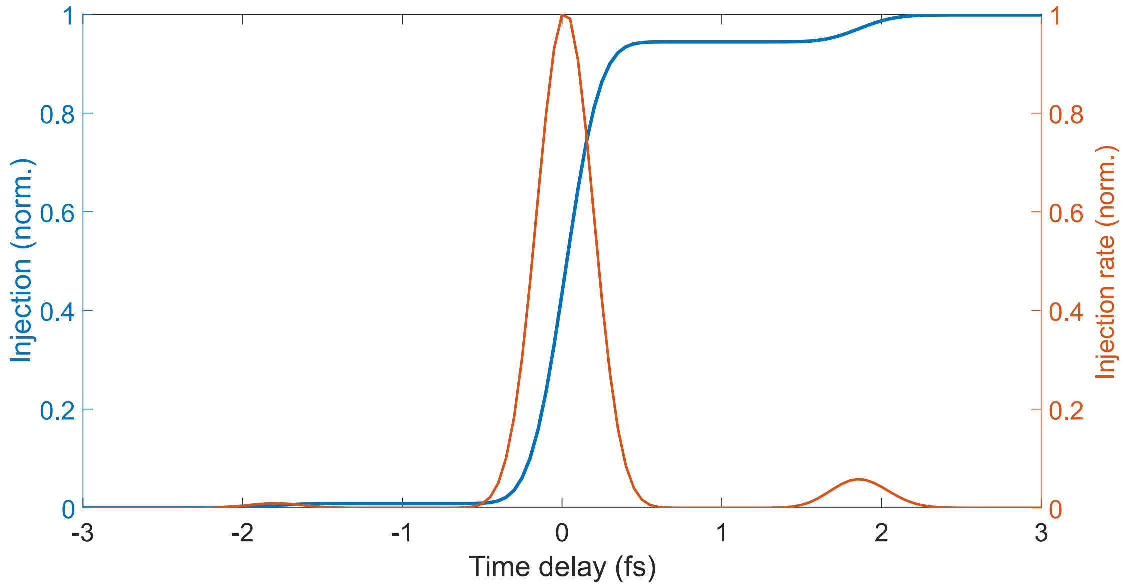

Figure 4S: Carrier injection rate of the synthesized pulse, which acts as the gate in NPS of the CH0 pulse.

To validate the pulse duration of CH0, retrieved via NPS, a cross-correlation frequency resolved optical gating (XFROG) measurement was also performed. This technique gives access to the group delay (GD) of a test pulse. To retrieve the GD of CH0, we perform frequency mixing with a narrowband spectral portion of CH2 (1500 ± 50 nm) in a 100 µm thick BBO crystal. The interaction of the two pulses results in the up-conversion of CH0. The spectrum of the up-converted pulse is

then recorded for different arrival times of CH0 relative to CH2 (Fig. 5Sa). The obtained GD curve is combined with the spectrum measured with a spectrometer. From this information, it is possible to calculate the intensity profile of CH0 after the crystal. To retrieve the field at the front surface of the crystal, the pulse is numerically back-propagated in the BBO to take into account the dispersion of the crystal. The calculated temporal evolution of the pulse is shown in Fig. 5Sc (blue line). The orange line indicates the Fourier-limited pulse (orange line). From the GD of CH0 a pulse duration of 2.4 fs (FWHM of the electric field squared) is obtained, in agreement with the NPS measurement (see Table 1 above). Using the field retrieved from Fig. 5Sa, and numerically mixing it with a narrowband pulse at 1500 ± 50 nm, it is possible to reconstruct the spectrogram shown in Fig. 5Sb. A good agreement between measured spectrogram (Fig. 5Sa) and reconstructed (Fig.5Sb) confirms the validity of the retrieved pulse. Despite providing similar information about the amplitude and phase of CH0, NPS yields higher resolution of the spectral phase due to highly nonlinear gating involved and long scan length in this case. Based on this fact, we employed the NPS measurement to calculate the spectral response of our EOS setup. The slight deviation between the pulse durations measured with two different techniques can be additionally explained by the retrieval and numerical backpropagation used for this implementation of XFROG.

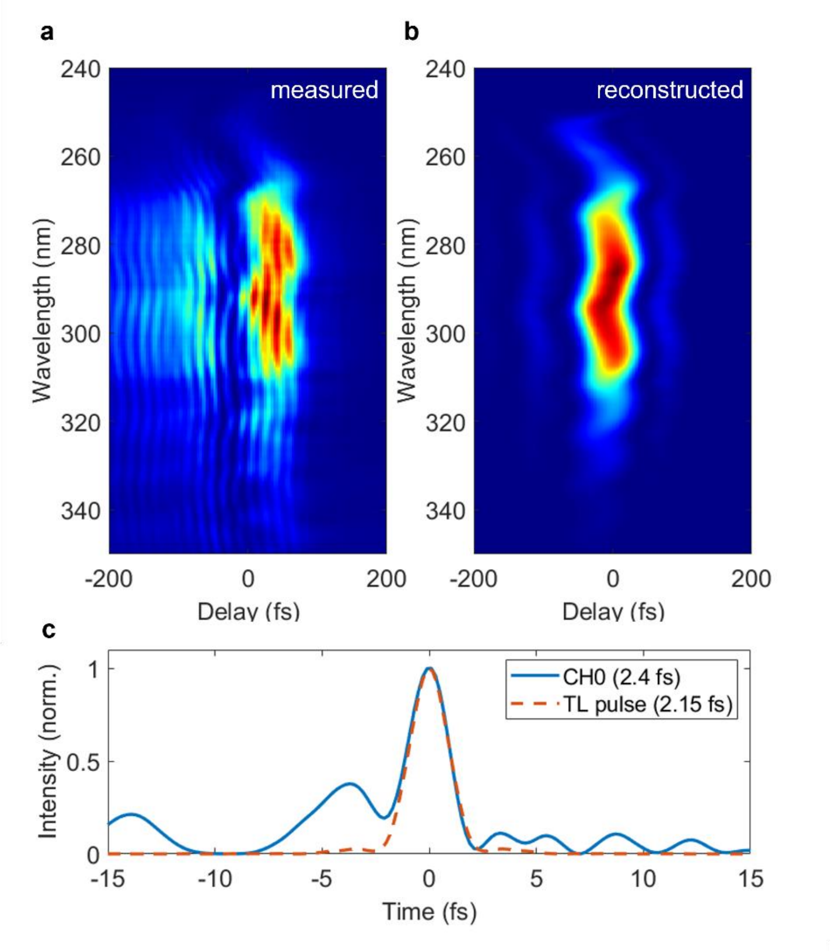

Figure 5S: a, Measured spectra as a function of time recorded to retrieve the GD of CH0. **b,** Reconstructed spectrogram obtained from **a. c**, Comparison between measured pulse envelope, in blue, and the envelope of its transform limited (TL) pulse, in orange.

EOS spectral response:

EOS directly provides the electric field of the test pulse only under the assumption that the sampling pulse is a delta function. If this is not the case, the electric field is a convolution with the response function of the measurement system. This implies that a deconvolution is necessary to retrieve the test pulse. The crystal and other components, furthermore, introduce an extra frequency-dependent term. The final form of the spectral response function, $S(\Omega)$, can be expressed in the following form

$$S(\Omega) = \int_{\Omega}^{\infty} d\omega_{LO} R(\omega_{LO}) |E(\omega_{LO})| |E(\omega_{LO} - \Omega)| e^{i[\phi(\omega_{LO}) - \phi(\omega_{LO} - \Omega)]} \quad (1)$$

Here, Ω is a sampled frequency that mixes with the probe frequency. The local oscillator frequency that interferes with the newly generated wave and its complex spectral amplitude are given by ω_{LO} and $|E(\omega_{LO})|e^{i\phi(\omega_{LO})}$, respectively. $R(\omega_{LO})$ is the product of the spectral response introduced by the crystal (type-I BBO, 10x6x0.005 mm, θ= 29.2°, φ= 90°), the band-pass filter (320 ± 30 nm), the Wollaston prism (uncoated MgF_2, 1° 20' Beam Separation) and the photodiodes (GaP, 3x3 mm^2).

The term $|E(\omega_{LO})|e^{i\phi(\omega_{LO})}$ defines the range of frequencies detectable with EOS. This depends on the bandwidth of the pulse, with a cut-off given by the difference in frequency between the highest and lowest frequency components present in the sampling pulse. In our case, where the sampling pulse spans 500 to 1000 THz, this leads to a cut-off of 500 THz as the theoretical limit of the response. The spectral amplitude and phase, as well as phase matching in the medium, impose additional constraints.

For a given sampling pulse, the other term that affects the response function is the contribution of the band-pass filter to $R(\omega_{LO})$. This has the effect of both increasing the SNR and of shifting to higher frequencies the detection bandwidth. This means that the spectral limit is higher than would be achievable if $R(\omega_{LO})$ was a constant. The interference between the sampling pulse and the up-converted test pulse can be detected as long as they overlap coherently on the photodiodes. As a result, the highest resolvable frequency (450 THz) is higher than the inverse of the pulse duration of the sampling pulse. The spectral filtering selects the portion of the light in which the high-frequency signals are most prevalent.

With the characterization performed via NPS (see main text), it is possible to calculate the spectral phase of CH0, our EOS sampling pulse. This information allows us to retrieve the electric field deconvolved by the spectral response of our setup and imperfection of CH0, the sampling pulse. All the traces shown in Fig. 2 and Fig. 3 of the main text have been corrected taking into account the calculated response function.

We calculate the full response function by solving the nonlinear wave equation in BBO. This includes the effects of dispersion of the local oscillator and phasematching in the build up of the nonlinear signal. The nonlinear wave equation in the slowly evolving wave approximation

$$\frac{\partial E_{\omega}(z)}{\partial z} = ik_{\omega} E_{\omega}(z) + \frac{i\omega}{2n(\omega)\epsilon_o c} P_{\omega}^{NL}(z), \quad (2)$$

where E_{ω} is a given spectral component of the field (all spectral components up to the Nyquist frequency of the time grid are included, and coupled via the nonlinear polarization), k_{ω} is the corresponding wavevector magnitude, $n(\omega)$ is the refractive index, and $P_{\omega}^{NL}(z)$ is the

corresponding spectral component of the nonlinear polarization, is solved numerically, using the measured (via NPS) sampling pulse, and a spectrally-flat field to be detected, for a series of delays between them. The phase and amplitude difference between the input field and measured waveform is therefore known for each frequency after Fourier transformation, as the input field is exactly known.

The form of the nonlinear polarization is relevant for such a calculation; this is done in the time domain, using the second order nonlinear tensor and the products of the fields on the axes of the crystal, in the usual way of the compact notation of the tensor. This implicitly assumes an instantaneous response of the nonlinear medium. Due to the narrow spectral filter in place in the measurement, and the fact that the sampling and measured fields do not overlap with resonances of the crystal, this is justified. However, it is useful to place limits distance from the resonances required before they begin to influence the measurement. This can be estimated as follows.

The simplest system that will show a second order nonlinear response is a two-level atom in the presence of a DC bias. If we describe the system with the time-dependent Hamiltonian

$$\hat{H} = \begin{pmatrix} \varepsilon_1 & \\ & \varepsilon_2 \end{pmatrix} + E(t)\begin{pmatrix} & \mu \\ \mu & \end{pmatrix}$$

where ε_1 and ε_2 are the energies of the ground and excited states, respectively, and μ is the dipole matrix element responsible for their coupling in the field $E(t)$, we can apply a static electric field E_{DC} in addition to the time-dependent electric field describing the laser pulses. Assuming that the field is switched on adiabatically, we can re-write the Hamiltonian as

$$\hat{H} = \begin{pmatrix} \varepsilon_1 & \mu E_{DC} \\ \mu E_{DC} & \varepsilon_2 \end{pmatrix} + E(t)\begin{pmatrix} & \mu \\ \mu & \end{pmatrix}$$

and work in a basis of the eigenstates of the matrix $\begin{pmatrix} \varepsilon_1 & \mu E_{DC} \\ \mu E_{DC} & \varepsilon_2 \end{pmatrix}$ at the start of the LHS. Starting with the initial wavefunction $\begin{pmatrix} 1 \\ 0 \end{pmatrix}$ in the diagonalized basis allows the influence of resonant effects such as the dispersion of the nonlinear susceptibility to be taken into account. As can be seen in Fig. 6S, the resulting EOS trace is nearly identical when calculated in this way, as compared to the assumption on an instantaneous polarization, when the energy difference, Δ, between the levels is set to 6.4 eV (the approximate band gap of BBO), with a delay of less than 0.01 attoseconds. In the case of a smaller gap (4.3 eV, similar to lithium niobate), a temporal shift of 136 attoseconds appears (it shifts to earlier delays because the effective probing event is later). Thus, in the present case, where all involved fields are far from crystal resonances, we expect the assumption of an instantaneous nonlinearity to be valid. This will not hold if either pulse overlaps with a resonance, of either the lattice or electronic system.

In the simulation a probe pulse of 2.5 fs duration and 450 nm central wavelength samples a field of 3 fs duration and 1 µm central wavelength, and the detected frequency band has a 50 THz width and 1 PHz central frequency. The signal is calculated through the time-dependent expectation value of the dipole operator over the course of the evolution of the wavefunction, as propagated via the time-dependent Schrödinger equation.

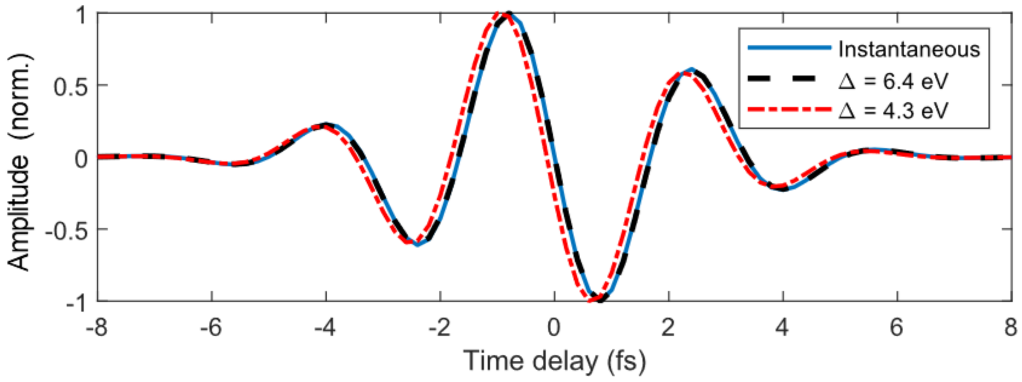

Figure 6S: Simulation of EOS in a two-level system. Depending on the band gap of the system relative to the probe pulse energy, a shift can appear due to resonant nonlinear effects. Involvement of the resonance results in the effective detection event taking place later, and thus shifting the waveform to earlier delays.

Data acquisition, time traces envelope and spectral limit:

The EOS traces have been acquired using a step size of 0.1 μm. The two traces reported below in Fig. 7S show the acquired raw data, without any data post-processing. The long-lasting oscillations in the case of CH2 (Fig. 7Sb) can be attributed to spectral components of the OPCPA that can be detected but not efficiently compressed.

From the raw data is possible to obtain the traces shown in Fig. 2 and 3 of the main text through two steps. First, a band-pass filter around the spectral region of interest is applied. This step removes artefacts that are not related to the test pulse fluctuations.

The second step implies correcting for the spectral response of the EOS setup. For further details see equation (1) above.

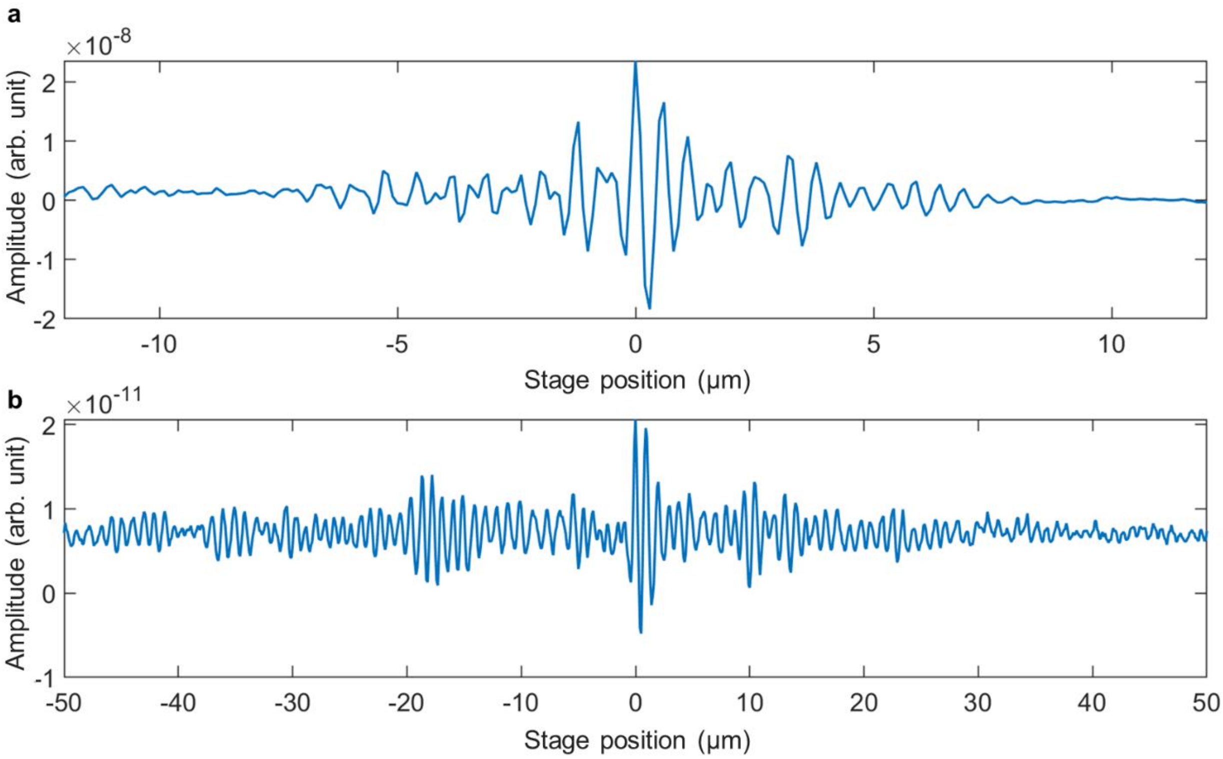

Figure 7S: Raw data acquired for CH1 (**a**) and CH2 (**b**), respectively.

After the data analysis the traces shown in Fig. 2 and 3 of the main text. The envelopes of the synthesized pulse and its two component channels are shown in Fig. 8. One can clearly see the shorter pulse duration of the synthesized pulse (yellow) compared to each channel taken separately (green for CH1 and red for CH2).

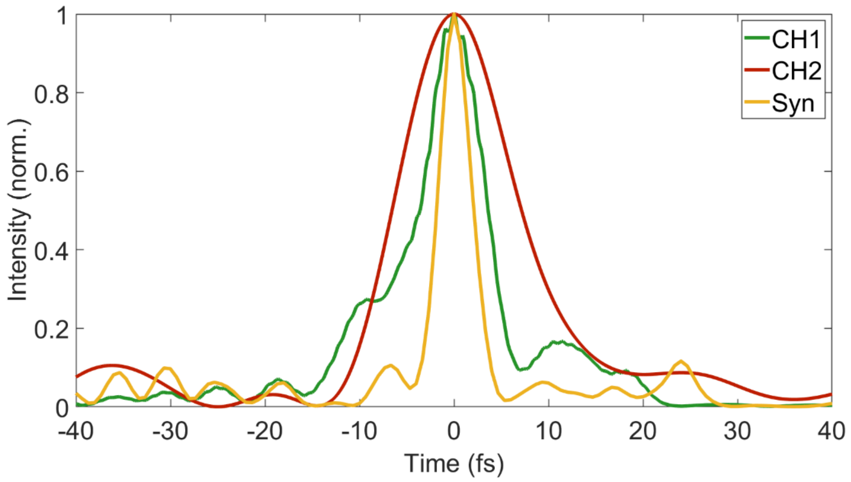

Figure 8S: Pulse envelope of the two synthesizer channels and of their combination.

By spectral filtering and averaging several time traces is possible to obtain the data shown in Fig. 2a and Fig. 2b. For CH1 it has been demonstrated in the main text that frequencies in the visible can be resolved. To confirm that these spectral components belong to the CH1 spectrum and are not an

artefact due to the data processing or the EOS detection, the FFT squared modulus of the EOS trace has been directly compared to the read out from a spectrometer (MayaPro2000, Ocean Optics). The two spectra are reported below in Fig. 9S. Spectral components beyond the working range of the chirped mirrors (700-1400 nm) have a modulated amplitude and are subject to strong dispersion that displaces much of the remaining energy outside of the temporal window of the time-resolved trace. Nonetheless, the signal is above the noise floor up to ~460 THz (652 nm).

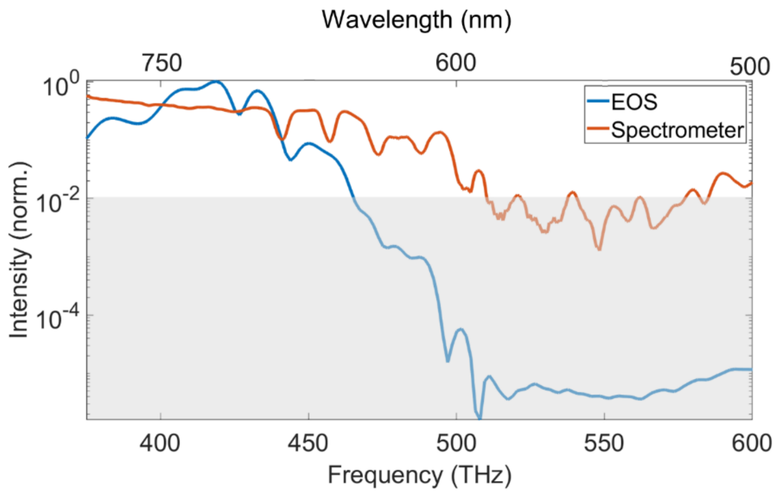

Figure 9S: Comparison between FFT squared of Fig. 2b and the spectrum recorded using a spectrometer, right before the EOS crystal. Both spectra show frequencies in the visible spectral region. The drop of the EOS spectrum at higher frequencies is due to the sampling pulse, CH0, not being compressed enough to sample such short frequencies. The CMs are not designed to compress the region above 400 THz, making this portion of the spectrum even harder to detect via a time-resolved method such as EOS.

Dispersion curves of the chirped mirrors

The measured dispersion curves of the chirped mirrors, which were manufactured in house, are shown in Fig 10S.

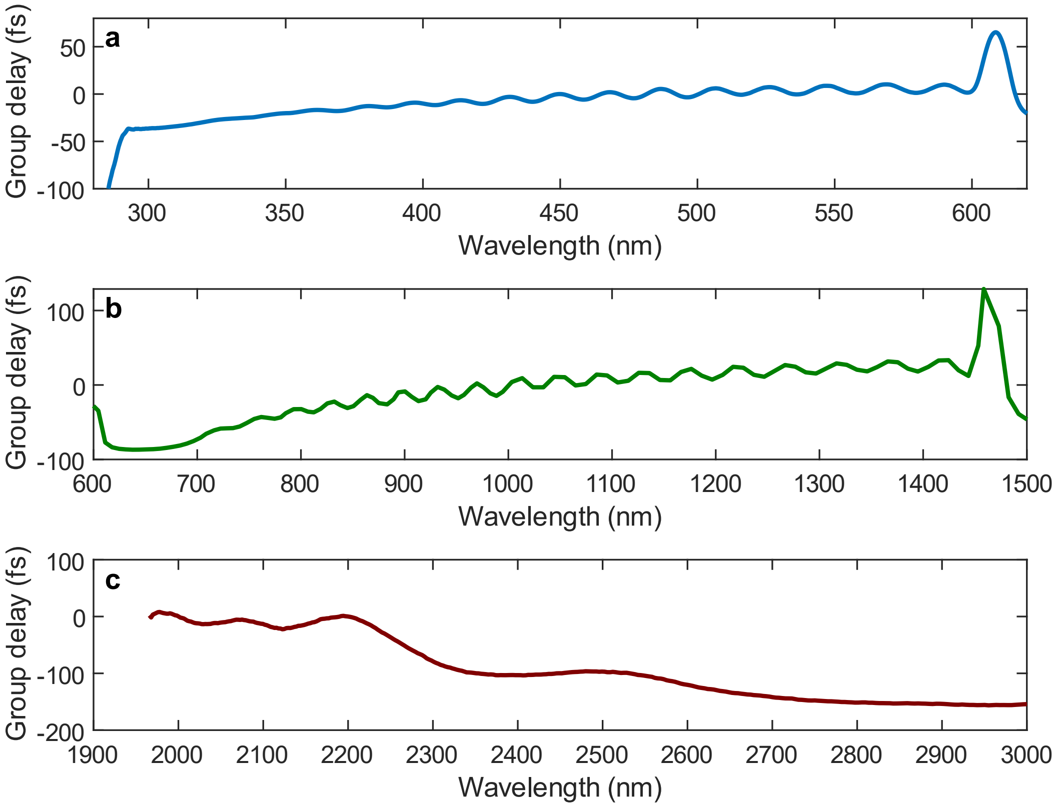

Figure 10S: Dispersion curves of the three channels. a CH0: Electro optic sampling pulse. **b** CH1: Short-wavelength synthesis channel. **c** CH2: long wavelength synthesis channel.

Development of EOS maximum frequency

As ultrafast technology has progressed, so has the maximum detectable frequency in EOS, from the initial demonstration of the sampling of free space THz beams, through the infrared. Some of these records are shown in Figure 11S.

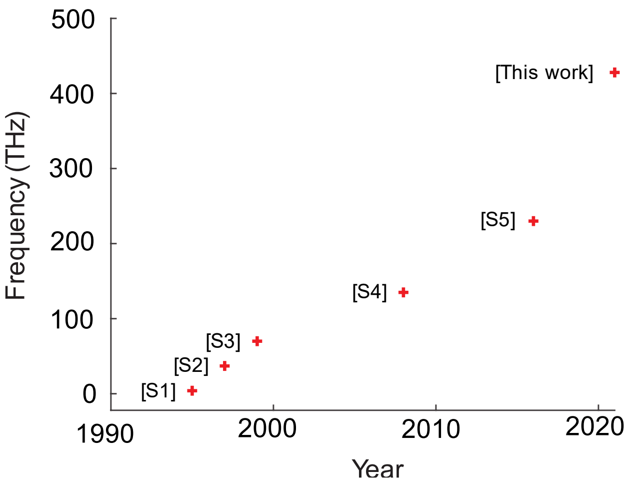

Figure 11S: Increase of maximum EOS frequency over time. References refer to the supplementary reference section.

Supplementary References

[S1] Wu, Q. and X.C. Zhang, *Free-space electro-optic sampling of terahertz beams.* Applied Physics Letters, 1995. **67**(24): p. 3523-3525.

[S2] Wu, Q. and X.-C. Zhang, *Free-space electro-optics sampling of mid-infrared pulses.* Applied physics letters, 1997. **71**(10): p. 1285-1286.

[S3]Leitenstorfer, A., et al., *Detectors and sources for ultrabroadband electro-optic sampling: Experiment and theory.* Applied physics letters, 1999. **74**(11): p. 1516-1518.

[S4] Sell, A., et al., *Field-resolved detection of phase-locked infrared transients from a compact Er: fiber system tunable between 55 and 107 THz.* Applied Physics Letters, 2008. **93**(25): p. 251107.

[S5] Keiber, S., et al., *Electro-optic sampling of near-infrared waveforms.* Nature Photonics, 2016. **10**(3): p. 159-162.
